# Lipid Metabolism and Relevance to Chronic Disease

**DOI:** 10.3390/nu17111936

**Published:** 2025-06-05

**Authors:** Harald Mangge

**Affiliations:** Clinical Institute for Medical and Chemical Diagnostics, Medical University of Graz, Auenbruggerplatz 15, A-8036 Graz, Austria; harald.mangge@medunigraz.at

Lipid metabolism is involved in the development and progression of widespread chronic diseases, making it a crucial area of study in medicine and public health [1]. Atherosclerosis, the major cause of cardiovascular disease (CVD), occurs by the accumulation of lipids, especially LDL cholesterol, in arterial walls, leading to plaque formation [2]. A chronic immune-mediated inflammation around the lipid core of the plaque can determine progression to life-threatening clinical events like myocardial infarction or stroke [2]. Therapies targeting lipid metabolism (e.g., statins, PCSK9 inhibitors) were found to significantly reduce morbidity and mortality from CVD [3], underlining the central role of dyslipidemia in CVD. Insulin resistance is also associated with dyslipidemia, particularly increased free fatty acids and fat accumulation in the liver. MAFLD, which stands for Metabolic (dysfunction)-Associated Fatty Liver Disease, results from excess lipid accumulation in liver cells. The LIFE study showed that MAFLD cases identified using the two fatty liver index (FLI) cut-off values were at higher risk for coronary artery and cerebrovascular diseases in an Asian (Japanese) population [4]. Additionally, a bidirectional relationship between periodontal disease and fatty liver may exist. MAFLD and periodontitis share common risk factors like obesity, insulin resistance, and dyslipidemia, which contribute to systemic inflammation [5]. In obesity, excess fat accumulation and dyslipidemia cause functional impairments in several metabolic pathways, both in adipose tissue and non-adipose tissues like liver, heart, pancreas, kidneys, and muscle [6]. Dyslipidemia is a driver of chronic low-grade inflammation in obesity [7]. The decline of regulatory T cells (T_regs_) in visceral tissue (VAT) in obesity leads to a disrupted VAT T_reg_ cholesterol homeostasis [8]. This observation suggests a bidirectional relationship between deregulation of the T-cell immune function and obesity-associated dyslipidemia [8]. Furthermore, lipid metabolism is involved in Alzheimer’s disease via ApoE4 variant-induced dyslipidemia [9]. This dyslipidemia may affect myelin integrity, inflammation, and neuronal health. In the case of cancer, altered lipid metabolism can increase cell division rates and growth-promoting cancer cell survival and metastasis [10]. In colorectal cancer, aberrant lipid metabolism is associated with Adenomatous Polyposis Coli (APC) tumor suppressor gene inactivation leading to activated Wnt/beta-catenin signaling for colorectal cancer initiation and progression [10].

To summarize, lipid metabolism is not only crucial for energy balance but is also deeply interconnected with the pathophysiology of many chronic diseases including the two most deadly ones, CVD and cancer.

This Special Issue addresses a broad spectrum of dyslipidemia-associated sequels. The contributions include the exploration of dietary supplements to improve lipoprotein composition [11,12], predictive value of lipoprotein profiles in diabetic retinopathy [13], association between cord blood lipidome and postnatal outcomes [14], and genetic aspects of adiponutrin polymorphisms involved in liver steatosis and atherosclerotic risk [15]. The contributions also address the effects of dyslipidemia on the prognosis of ulcerative colitis [16], omega 3 fatty acids and breast cancer [17], the interactions of lipids with osteoarthritis [18], and the effects of adipose tissue distribution on ostecalcin [19]. Finally, perspectives on lipidomics in NAFLD [20] and the controversial role of homocystein and vitamin B deficiency in CVD are critically discussed [21].
